# Supplementary material for: Long-Term Evaluation of Intranigral Transplantation of Human iPSC-Derived Dopamine Neurons in a Parkinson’s Disease Mouse Model
Source: Cells. 2022 May 10;11(10):1596. doi: 10.3390/cells11101596 (PMC9140181; doi:10.3390/cells11101596)
Supplement: Supplementary file 1 [file cells-11-01596-s001.zip › Supplemental data.pdf]

Antibodies	Sources	References	Dilutions
**Rabbit anti-Cleaved Caspase-3**	Cell Signalling	9664	1/200
**Rabbit anti-Calbindin D-28K**	Swant	CB-38a	1/500
**Goat polyclonal anti-DCX (C-18)**	Santa Cruz	sc-8066	1/250
**Rat monoclonal anti-Dopamine Transporter**	Abcam	Ab5990	1/200
**Rabbit anti-En2**	Abcam	Ab28731	1/500
**Mouse monoclonal anti-FoxA2**	Abcam	60721	1/500
**Rabbit monoclonal anti-FoxA2**	Abcam	ab108422	1/500
**Mouse monoclonal anti-GAD67**	Millipore	MAB5406	1/500
**Chicken polyclonal anti-GFAP**	Abcam	Ab4674	1/500
**Goat polyclonal anti-Girk2**	Abcam	Ab65096	1/200
**Rabbit anti-Ki-67**	Abcam	Ab16667	1/500
**Rabbit polyclonal anti-Lin28**	Abcam	Ab46020	1/500
**Rabbit polyclonal anti-Lmx1A**	Abcam	Ab139726	1/500
**Rabbit polyclonal anti-Nanog**	Abcam	Ab80892	1/150
**Mouse monoclonal anti-hNCAM (ERIC1)**	Santa Cruz	sc-106	1/200
**Mouse monoclonal anti-Nestin**	Abcam	ab6320	1/200
**Mouse anti-NeuN**	Millipore	MAB377	1/200
**Rabbit anti-NeuN**	Abcam	177487	1/200
**Mouse monoclonal anti-Human Nuclear Antigen**	Abcam	Ab191181	1/200
**Mouse monoclonal anti-Nurr1**	Abcam	Ab41917	1/200
**Rabbit polyclonal anti-Oct4**	Abcam	Ab18976	1/500
**Rabbit polyclonal anti-Olig2**	Millipore	AB9610	1/500
**Goat polyclonal anti-Otx2**	R&D	AF1979	1/200
**Rabbit anti-Human Pitx3**	ThermoScientific	701181	1/200
**Goat polyclonal anti-Serotonin**	Abcam	Ab66047	1/200
**Rabbit polyclonal anti-SOX2**	Abcam	Ab97959	1/250
**Mouse monoclonal anti-SSEA1**	Abcam	Ab16285	1/100
**Mouse monoclonal anti-SSEA4**	Abcam	Ab16287	1/200
**Chicken polyclonal anti-TH**	Abcam	Ab76442	1/1000
**Mouse monoclonal anti-TH**	Immunostar	22941	1/5000
**Rabbit anti-TH**	Cell Signaling	2792	1/500

Supplemental Figure S1: Primary antibodies used in the study. The table shows the antigen, host and dilution used for each immunofluorescence staining in the study. The company and catalogue numbers are also included.

Antibodies	Source	Reference	Dilution
**Donkey-anti Chicken Alexa Fluor 488**	Jackson ImmunoResearch	703-545-155	1/500
**Donkey-anti Chicken FluoProbes 547**	Interchim	FB-SB1110	1/500
**Donkey-anti Chicken Alexa Fluor 647**	Jackson ImmunoResearch	703-605-155	1/500
**Donkey anti-hicken Alexa Fluor 790**	Jackson ImmunoResearch	703-655-155	1/500
**Donkey-anti Goat Alexa Fluor 488**	LifeTechnologies	A11055	1/500
**Donkey-anti Goat Alexa Fluor 568**	LifeTechnologies	A11057	1/500
**Donkey-anti Goat Alexa Fluor 647**	LifeTechnologies	A21447	1/500
**Donkey anti-Goat Alexa Fluor 790**	Jackson ImmunoResearch	705-655-147	1/500
**Donkey anti-Mouse Alexa Fluor 488**	LifeTechnologies	A21202	1/500
**Donkey anti-Mouse Alexa Fluor 568**	LifeTechnologies	A10037	1/500
**Donkey anti-Mouse Alexa Fluor 647**	LifeTechnologies	A31571	1/500
**Donkey anti-Mouse Alexa Fluor 790**	Jackson ImmunoResearch	715-655-151	1/500
**Donkey-anti-Rabbit Alexa Fluor 350**	LifeTechnologies	A10039	1/500
**Donkey-anti-Rabbit Alexa Fluor 488**	LifeTechnologies	A21206	1/500
**Donkey-anti-Rabbit Alexa Fluor 568**	LifeTechnologies	A10042	1/500
**Donkey-anti-Rabbit Alexa Fluor 647**	LifeTechnologies	A31573	1/500
**Donkey anti-Rabbit Alexa Fluor 790**	Jackson ImmunoResearch	711-655-152	1/500
**Donkey anti-Rat Alexa Fluor 488**	LifeTechnologies	A21208	1/500
**Donkey anti-Rat FluoProbes 547**	Interchim	FP-SB6120	1/500
**Donkey anti-Rat Alexa Fluor 647**	Jackson ImmunoResearch	712-606-153	1/500
**Donkey anti-Rat Alexa Fluor 790**	Jackson ImmunoResearch	712-655-153	1/500

Supplemental Figure S2: Secondary antibodies used in the study. The table shows the antigen, host and dilution used for each immunofluorescence staining in the study. The company and catalogue numbers are also included.

Supplemental Video S1: Video showing hNCAM/TH+ fibers along the nigrostriatal pathway in a cleared mouse hemisphere, 1 year after transplantation into the SNpc (in .mp4 file, Supplementary to Figure 8). This acquisition was performed using 4X magnification, followed by reconstruction of the mosaic using Terastitcher. In this movie, we can see many fibers from the graft located at the level of the ventral midbrain, following the mfb to the striatum. A large part of the fibers is hNCAM+ (red) / TH+ (green).

Supplemental Video S2: Video showing hNCAM/TH+ fibers in the CPu in a cleared mouse hemisphere, 1 year after transplantation into the SNc (in .mp4 file, Supplementary to Figure 8). This acquisition was performed using 4X magnification, followed by reconstruction of the mosaic using Terastitcher. In this movie, we can see that part of the hNCAM+ fibers (in red) have reached their target structure: the striatum. Most of these fibers are TH+ (in green).
